# Juvenile ossifying fibroma in anterior ethmoidal sinus in B-cell acute lymphoblastic leukemia and MHC class II deficiency patient: Case report

**DOI:** 10.1016/j.ijscr.2023.108133

**Published:** 2023-04-07

**Authors:** Abdulaziz Ali Alnasser, Abdurhman Alsaif, Ali H. Alassiri, Mazyad M Alenezi, Jaber Alshammari

**Affiliations:** a College of Medicine, King Saud bin Abdulaziz University for Health Sciences, Riyadh, Saudi Arabia; b Division of Anatomical Pathology, Department of Pathology & Laboratory Medicine, King Abdulaziz Medical City, Ministry of National Guard - Health Affairs, Riyadh, Saudi Arabia; c Department of Otolaryngology Head and Neck Surgery, College of Medicine, Qassim University, Qassim, Saudi Arabia; d Department of Pediatric Surgery, Ministry of the National Guard - Health Affairs, Riyadh, Saudi Arabia; e Department of Pediatric Surgery, King Abdullah International Medical Research Center, Riyadh, Saudi Arabia; f Department of Pediatric Surgery, King Saud bin Abdulaziz University for Health Sciences, Riyadh, Saudi Arabia

**Keywords:** Juvenile ossifying fibroma, Acute lymphoblastic leukemia, Ethmoidal sinus, Case report

## Abstract

**Introduction and importance:**

Juvenile ossifying fibromas (JOF) are rare benign tumors affecting the craniofacial area, and they present more in younger age groups. JOFs are aggressive lesions and have a high tendency for recurring after surgical resection.

**Case presentation:**

A 9-years-old female who was a known case of major histocompatibility complex class II deficiency since birth and post hematopoietic stem cell transplant was diagnosed with B-cell acute lymphoblastic leukemia (ALL). Right anterior ethmoidal sinus opacification with mucosal thickening found on sinus imaging was initially thought to be infectious in origin due to the patient's immunodeficiency. However, further investigation and endoscopic sinus surgery with lesion debridement and biopsy revealed psammomatoid JOF. Follow up and imaging results urged the need for a second surgery, but no recurrence was detected. As the patient's ALL was being treated, the case was followed up for two months with imaging showing opacification in multiple sinuses which was suspected to be fungal infection due to immunodeficiency. The complicated character of the case may have hindered any additional invasive management. Opacification persisted despite conservative management. Unfortunately, the patient suffered from disease complication, infections, and died from multi-organ failure.

**Clinical discussion:**

This case highlights the importance of a thorough diagnostic workup and management strategy for immunodeficient patients with JOF. JOF's recurrent nature along with the pre-existing immunodeficiency made this case difficult to manage as the patient had other life-threatening condition.

**Conclusion:**

Specific management considerations should take place in immunocompromised patients. Post-operative long-term follow-ups are needed for early detection of recurrence.

## Highlights

• Juvenile ossifying fibromas (JOF) are rare benign tumors affecting the young and most require surgery.
• It is difficult to ensure complete removal of lesions near the skull base.
• Specific management considerations should take place in immunocompromised patients as they are at high risk of infections that may be confused with JOF recurrence.
• Our case together with another case in the literature both reported patients with JOF and acute lymphoblastic leukemia.

## 1 Introduction

Ossifying fibroma (OF) is a benign fast-growing tumor that replaces normal bone with fibrous tissue containing a newly formed mineralized product [1], [2]. Clinicopathologically, OF is divided into cemento-osseous OF (COF) and Juvenile OF (JOF) with its two subtypes, psammomatoid and trabecular [3]. JOF has been distinguished from COF based on age, site predilection, and clinical behavior. JOF is an aggressive rapidly growing benign tumor that primarily affects children with the craniofacial area being the most affected. It also has a high tendency for recurrence [4]. On the other hand, COFs are usually less aggressive and have an increased incidence among patients in their third and fourth decades of life [5].

We are presenting a case of juvenile psammomatoid ossifying fibroma (JPOF) found in the right anterior ethmoidal sinus of a 9-years-old female who was recently diagnosed with B-cell acute lymphoblastic leukemia (ALL) and is a known case of major histocompatibility complex class II (MHC2) deficiency. The patient was managed in King Abdullah Specialized Children's Hospital which is a governmental tertiary hospital in Riyadh, Saudi Arabia. The work reported in this case study has been conducted in accordance with the Surgical case Report (SCARE) guidelines [6].

## 2 Presentation of case

The 9-year-old female was a known case of MHC2 deficiency with a family history of a previously affected sibling with the same condition. The diagnosis was confirmed by genetic study with homozygous mutation on RFXANK gene. Since birth, the patient has been following up in the immunology clinic. The patient had a hematopoietic stem cell transplant (HSCT) at the age of 5, for which the patient required regular visits for intravenous immunoglobulin transfusion.

**Fig. 1:**
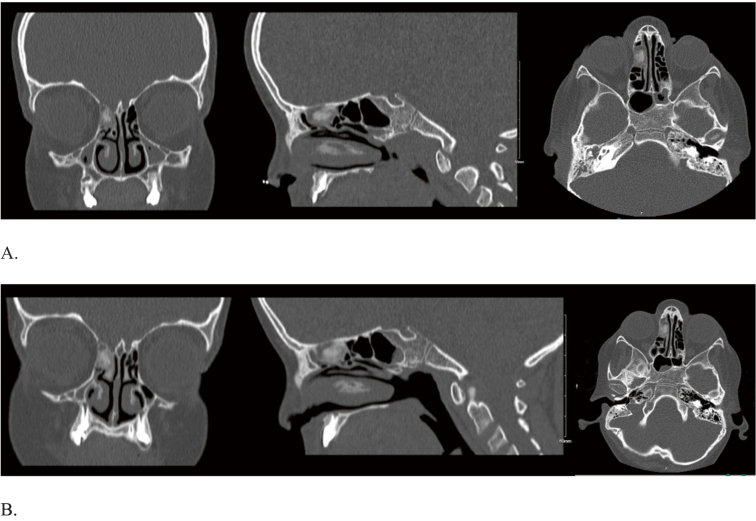
A) Different views of the sinus CT scan at the first presentation showing right anterior ethmoidal sinus complete hyperdense opacification. B) Different views of the second sinus CT after antimicrobial management showing the persistence of the right anterior ethmoidal sinus complete hyperdense opacification.

At one visit, the patient had low grade fever which urged for more detailed history. The patient complained of right ear otalgia with no discharge, decreased appetite, weight loss, and lower abdominal pain that lasted for a week. Antibiotics were given for suspected acute otitis media, and complete blood count results showed a picture of leukemia. After full hematologic workup, the patient was diagnosed with B-cell ALL with BCR-ABL rearrangement and monosomy 7. The patient complained of an intermittent frontal headache. Sinus computed tomography (CT) showed complete opacification of the right middle ear, mastoid air cells, mucosal thickening of the bilateral maxillary and sphenoid sinuses, and right anterior ethmoidal air cells complete hyperdense opacification, suggestive of fungal infection (Fig. 1A). The pediatric otolaryngology-head & neck surgery (OHNS) team was consulted to see the patient. The patient denied any nasal symptoms apart from frontal headache. On examination, the otoscope showed intact bilateral tympanic membrane, the nasal scope showed bilateral intact nasal cavity with no discharge, polyps, eschar, or necrosis, and clear throat exam. Nasal swabs were done for fungal and bacterial culture.

Due to immunodeficiency, empiric antifungal agent was administered while fungal and bacterial stain and culture results were pending. The microbiological culture showed a growth of methicillin-sensitive *Staphylococcus aureus* with no fungal growth. The patient was given antibiotics and continued antifungal treatment. Symptoms persisted after the antimicrobial management, and another sinus CT demonstrated the persistence of the hyperdense opacification in the right anterior ethmoidal sinus (Fig. 1B).

**Fig. 2:**
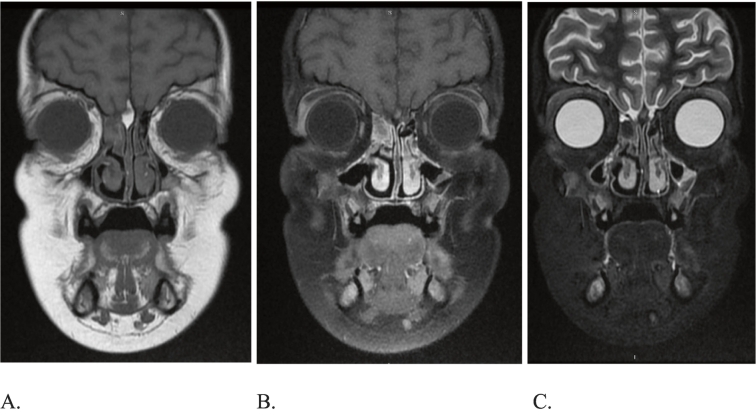
Right anterior ethmoidal sinus lesion on coronal view of sinus MRI. A: Low signal intensity on T1. B: No significant enhancement on T1 with contrast and fat suppression. C: Low signal intensity on T2.

Sinus contrasted magnetic resonance imaging (MRI) showed finding consistent with fungal sinusitis with no intraorbital or intracranial extension (Fig. 2).

**Fig. 3:**
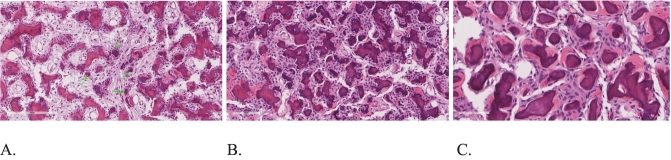
Hematoxylin and Eosin-stained sections show a fibro-osseous lesion with frequent psammomatoid cementum droplets and in-between bland spindle cell proliferation. Trabecular woven bone rimmed by osteoblasts is also observed. No overt malignant features seen. A: Low power view showing the fibrous and osseous components as well as the cementum droplets (green arrows). B & C: Intermediate power views depicting the frequent psammomatoid cementum droplets characteristic of Juvenile psammomatoid ossifying fibroma.

Endoscopic sinus surgery (ESS) was performed under general anesthesia by the pediatric OHNS team. The aim of surgery was lesion debridement and to obtain microbiological and histopathological samples. Nasal cavity examination showed normal findings. Anterior ethmoidectomy showed friable bony septation that was entirely removed up to the frontal recess. Specimens were collected from the right anterior ethmoidal sinus for histopathology analysis, fungal, bacterial, tuberculosis cultures and stains. The histopathology report revealed JPOF (Fig. 3).

**Fig. 4:**
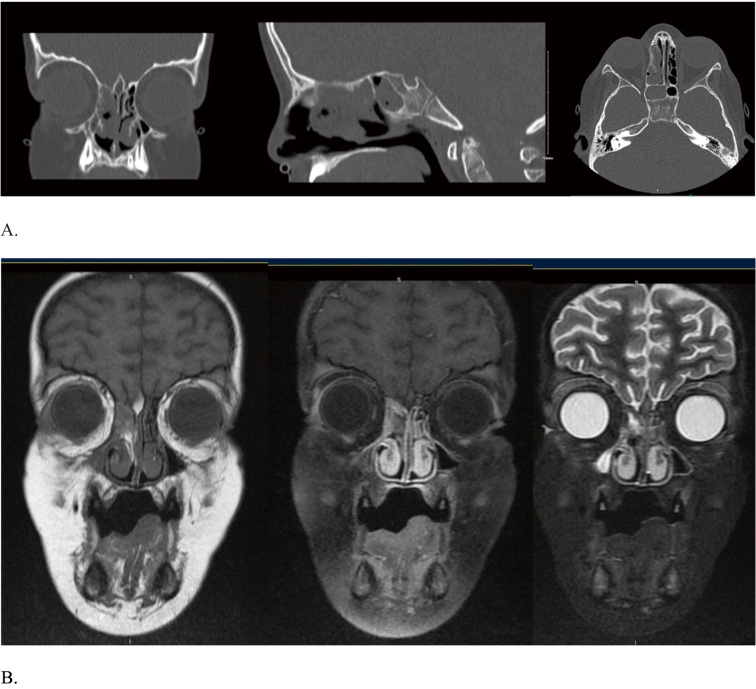
A) After the first surgery, regression of the previously visualized right anterior ethmoidal hyperdense opacification with residual peripheral hyperdense opacification was seen in different views. B) MRI with contrast prior to the second surgery showing low signal intensity on T1 (left), contrast and fat suppression on T1 (middle), and high signal intensity on T2 (right). The right anterior ethmoid sinuses showing residual mucosal thickening and sinus fluid, and bilateral opacification of the maxillary sinuses with low intensity in T1 (Left), minimal enhancement post contrast more in the right side and high intensity in T2 (right).

Follow up CT imaging after the surgery showed interval regression of the previously visualized right anterior ethmoidal hyperdense opacification with residual peripheral hyperdense opacification and rarefaction of the right lamina papyracea with no obvious intraorbital extension. In addition, there was interval worsening of previously visualized mucosal thickening within maxillary, ethmoid, and sphenoid sinuses more prominently on the right side which was becoming denser than before. Also, osteomeatal complexes were occluded bilaterally (Fig. 4A). Additional contrasted MRI was done for further evaluation (Fig. 4B). ESS with nasal debridement was performed and nasal biopsy was done to rule out invasive fungal infection and JOF recurrence. During the procedure, examination of the nose revealed yellowish discharge on the right side scattered with debris that could be from a previous hemostatic dissolvable agent. Right maxillary antrostomy and sphenoid sinuses were opened, and no abnormalities were identified. Multiple biopsies were taken from the right ethmoidal sinus and the right middle turbinate. The histopathology showed granulation tissue and fibrin which were negative for fungal hyphae.

**Fig. 5:**
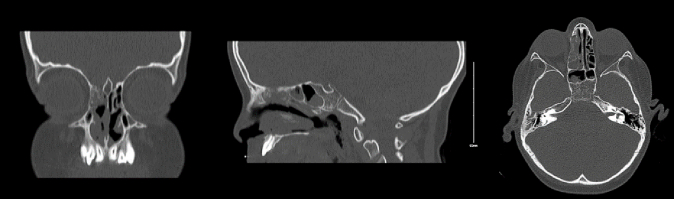
Interval progression of right anterior ethmoid opacification was noticed in different views.

After several weeks, patients complained of headache with no visual disturbances. Sinus CT showed redemonstration of partial opacification right ethmoid air cell, right maxillary air cells, and right sphenoid air cells that raise suspicion for fungal infection even though the patient was on prophylactic antifungal treatment (Fig. 5). Bedside flexible examination showed no discharge or masses in nasal cavity. So, the patient was managed conservatively.

The patient had second allogenic HSCT from a 1st degree family member. Afterwards, the patient developed febrile neutropenia that urged additional work up. CT sinus showing unchanged finding from previous scans. The patient's health deteriorated and diagnosed with Engraftment syndrome. Patient had respiratory distress and septicemia. Unfortunately, the patient developed multi-organ failure and died.

## 3 Discussion

Fibro-osseous lesions of the head and neck can vary from simple benign slowly growing lesions to locally aggressive, disabling, and deforming disease [7]. Histopathological features of JOF vary depending on the subtype, and each subtype favors different locations [7], [8].

Histologically, the trabecular variant is characterized by a cell-rich fibrous stroma that contains spindle cells which produce little amount of collagen. The osteoid arises directly from the stroma and grows to form long thin threads that undergo an irregular mineralization process at their centers and ultimately develop into woven bone trabeculae lacking osteoblastic rimming [5], [7], [8]. Moreover, the trabecular variant is seen most in the maxilla. On the other hand, the psammomatoid variant mostly arises in the orbital and paranasal sinuses' bony walls. Microscopically, JPOF exhibits numerous small round psammomatoid body-like ossicles in a fibroblastic stroma with variable cellularity. The ossicles somewhat resemble the dental cementum [7], [8], [9].

Among imaging modalities used to evaluate JOF, CT and MRI can be utilized. Based on a previous study, the use of CT alone as a diagnostic tool is sufficient for the evaluation of JOF [10]. Moreover, the presence of demarcation can distinguish JOF from other differentials such as fibrous dysplasia. Radiologically, JOF is characterized as an expansive single lesion with well-defined peripheral borders, no soft tissue involvement, and variable internal structure opacification which allows for the differentiation between JOF subtypes. JPOF is a ground-glass opaque lesion that can present in three ways; either as a thick cortex with a central radiolucent core, a solitary ground-glass mural core, or a solid homogeneous radio-opacity. In contrast, JTOF is essentially radio-transparent with irregular and dispersed calcification [11]. In our case, imaging findings were initially thought to be infectious in origin due to the patient's complex history of immunodeficiency and chronic steroid therapy after HSCT.

Our patient was incidentally found to have B-cell ALL, and then was diagnosed with JPOF. A similar case reported in the literature of a female patient who had ALL and was in remission for 3 years. This patient presented with a tumor in the right ramus and corpus of the mandible causing facial asymmetry, and a pathology report confirmed the diagnosis of JOF [5]. This case together with ours may have had the two same conditions coincidentally; however, this also can raise suspicion for an undiscovered relationship between ALL and JOF.

Due to the aggressiveness of the lesion and its high recurrence rates, management approach for such lesions can range from complete surgical resection, enucleation only, enucleation followed by curettage or peripheral ostectomy, or curettage only [12]. The choice of the surgical approach depends on the type, location, extent, aggressiveness of the lesion [12], and the anatomical relation to the adjacent structures [13]. Furthermore, a recent systematic review demonstrated that enucleation alone has the highest recurrence rate, but the rate will be lower if enucleation is done with either curettage or peripheral ostectomy [12]. Even though complete surgical resection of JOF has near absent recurrence, enucleation followed by either peripheral ostectomy or curettage should be the mainstay approach for the variants of JOF to avoid the unwanted deformities associated with resection surgeries [5], [14]. Moreover, the complete removal of the lesion in our case could not be ensured due its location as it was closely related to the skull base. Thus, recurrence was highly suspected after the first surgery. Post-operative long-term follow-ups are needed for early identification of any recurrence.

## 4 Conclusion

Juvenile ossifying fibroma is a rare clinicopathological entity that mainly affects the young. There may be a relationship between JOF and ALL, and specific considerations for the management should take place in immunocompromised patients. Post-operative long-term follow-ups are needed for early detection of recurrence.

## Parental consent for minors

Written informed consent was obtained from the patient's parents/legal guardian for publication of this case report and accompanying images. A copy of the written consent is available for review by the Editor-in-Chief of this journal on request.

## Ethical approval

Ethical approval is exempt/waived at our institution.

## Funding

This research did not receive any specific grant from funding agencies in the public, commercial, or not-for-profit sectors.

## Guarantor

Dr. Mazyad Alenezi.

## Research registration number

Not applicable.

## CRediT authorship contribution statement

Jaber Alshammari: concept, supervision.
Abdulaziz Ali Alnasser: drafting, literature review, writing.
Abdurhman Alsaif: drafting, literature review, writing.
Mazyad Alenezi: supervision, critical review, drafting.
Ali H. Alassiri: writing, histopathology slides review.

## Conflict of interest

All the authors declare no conflict of interest and have no financial or personal gains and benefits from this work.
